# A Case Report on Detecting Porcelain Gallbladder form Wall-Echo-Shadow Sign on Point-of-Care Ultrasound

**DOI:** 10.21980/J8164G

**Published:** 2021-04-19

**Authors:** Fares Al-Khouja, Proma Mazumder, John Moeller, Shadi Lahham

**Affiliations:** * University of California, Irvine, School of Medicine, Irvine, CA; ^ Touro University Nevada, College of Osteopathic Medicine, Henderson, NV; † University of California, Irvine, Department of Emergency Medicine, Orange, CA

## Abstract

Point-of-Care Ultrasound (POCUS) is a rapidly growing means of accelerated diagnosis for various conditions. This case report demonstrates POCUS use to differentiate porcelain gallbladder (PGB) from wall-echo-shadow (WES) sign.

A 75-year-old female presented for evaluation of elevated liver function tests (LFTs). Upon arrival, she had no acute complaints with unremarkable vital signs, aside from elevated blood pressure of 155/79 mmHg. Review of LFTs demonstrated elevation of total bilirubin, aspartate aminotransferase (AST), alanine aminotransferase (ALT), and alkaline phosphatase (ALP). Physical examination and review of systems were unremarkable. POCUS revealed common bile duct dilatation with cholelithiasis and sludge. While these findings may be confused with a WES sign, POCUS also revealed a hyperechoic gallbladder wall (GBW) with shadowing and irregular clumps of echo. The hyperechoic GBW with irregular shadowing heightened the suspicion for PGB. Computed tomography scan confirmed the presence of PGB. The patient had a stent placed near the common hepatic duct stricture and was advised to return for cholecystectomy. She was readmitted three months later due to bacteremia, likely caused by gallbladder obstruction in the setting of gallbladder cancer.

Point of care ultrasound can be used to identify PGB. Because PGB has been associated with gallbladder carcinoma, prompt diagnosis is vital in early and aggressive treatment. In this case report, we demonstrate a reliable method in properly diagnosing PGB through POCUS.

**Topics:**

Point-of-care ultrasound, ultrasound, porcelain gallbladder, WES sign, wall-echo-shadow sign.[Fig f1-jetem-6-2-v25][Fig f2-jetem-6-2-v25][Fig f3-jetem-6-2-v25]

**Figure f1-jetem-6-2-v25:**
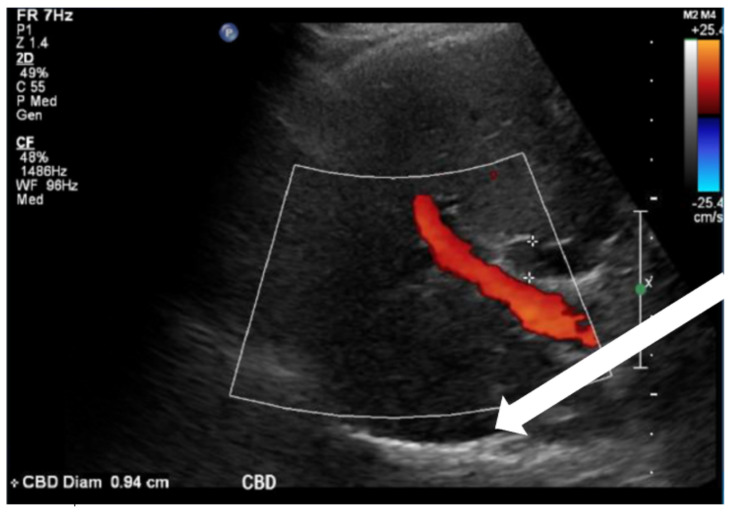


**Figure f2-jetem-6-2-v25:**
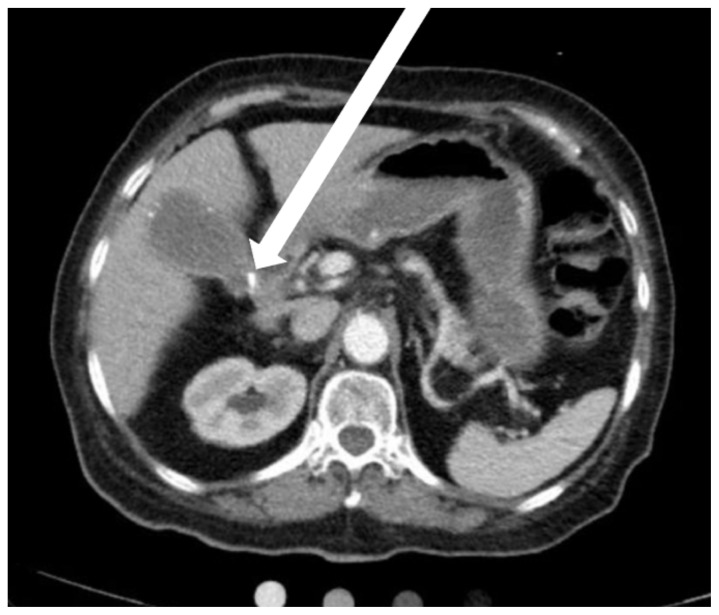


**Figure f3-jetem-6-2-v25:**
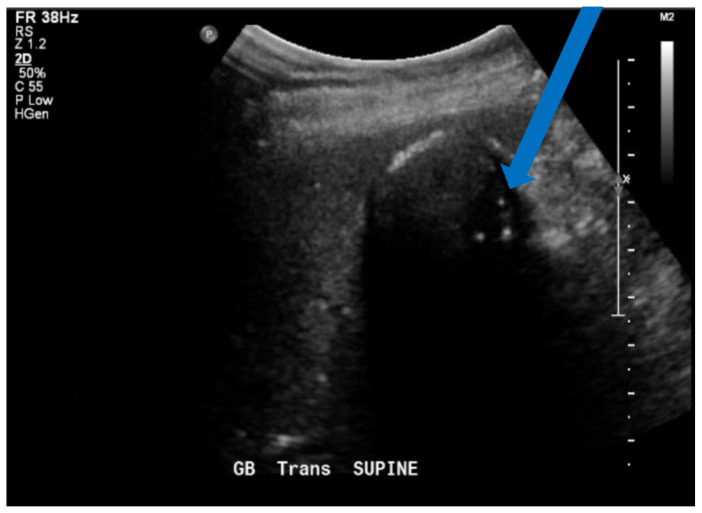


## Brief introduction

Porcelain gallbladder (PGB) is a rare condition that causes bluish discoloration and either complete or incomplete calcification of the gallbladder wall.^1^ Porcelain gallbladder may cause symptoms such as abdominal pain, nausea, vomiting, diarrhea, jaundice, and anorexia. However, the majority of cases are asymptomatic.^2^ The etiology and mechanism by which PGB advances is not completely understood but is thought to result from chronic cholecystitis that stems from long term inflammation. In fact, 90–95% of all PGB cases are associated with cholelithiasis.^1^ The incidence of PGB is predominantly in elderly women 50–70 years of age, with an overall incidence of 0.06–0.8%. Rapid identification of PGB is vital due to a concern for the development of malignancy. Anywhere between 12.5–62% of patients with a PGB diagnosis will develop gallbladder carcinoma.^3^ PGB is suggested to be associated with gallbladder carcinoma, especially infiltrating adenocarcinomas.^4^ On ultrasound, PGB can produce similar ultrasonographic findings as wall-echo-shadow (WES) sign. A WES sign is indicative of cholelithiasis that has progressed to fill the entire lumen of the gallbladder.^5^ In this report, we demonstrate the use of point-of-care ultrasound (POCUS) in the diagnosis of PGB and how to distinguish PGB from WES sign in a female presenting with concerning bloodwork results from her primary care provider.

## Presenting concerns and clinical findings

A 75-year-old female with no known history of gallbladder disease presented to the emergency department (ED) after obtaining routine blood work from her primary care provider that revealed elevated liver function tests. Upon arrival to the ED, the patient did not have any complaints of acute symptoms, including fever, abdominal pain, nausea, vomiting, or diarrhea. Outpatient labs were notable for a total bilirubin of 1.7 μmol/L, Aspartate Aminotransferase (AST) of 224 U/L, Alanine Aminotransferase (ALT) of 204 U/L, and alkaline phosphatase (ALP) of 904 IU/L. Repeat labs upon arrival to ED showed total bilirubin of 2.2, AST of 227, ALT of 230, and ALP 931. On history, the patient reports she had recently returned from travel to Mexico within the previous four months. Initial triage vitals were largely unremarkable, with a heart rate of 81 beats per minute, temperature of 99.1 degrees Fahrenheit, and 16 respirations per minute. However, she had an elevated blood pressure of 155/79 mmHg. Physical examination was unremarkable with normal review of systems and no abdominal tenderness, guarding, or rebound.

## Significant findings

Point-of-care ultrasound (POCUS) was performed by the emergency physician. Gallbladder ultrasound (US) should be performed using a curvilinear probe. If the patient's body habitus does not allow for the use of a curvilinear probe, a phased array probe may be used. To find the gallbladder with ultrasonography, two approaches are commonly used. Many physicians prefer the “subcostal sweep” in which the probe is placed on the xiphoid process in a sagittal plane and swept along the inferior costal margin until the gallbladder is visualized. If this does not adequately locate the gallbladder, the “X minus 7” approach may be used. In this approach, the probe is placed on the xiphoid (X) process in a transverse view and moved 7 centimeters (minus 7) to the patient's right. This technique is useful for patients with a larger body habitus. If the gallbladder is still not visualized, placing the patient in left lateral decubitus position or asking them to take a deep breath and hold may help the ultrasonographer locate the gallbladder. The US revealed mild hepatic biliary duct dilation with cholelithiasis and sludge, but no additional evidence to suggest cholecystitis. The US image showed a dilated common bile duct at 0.94 cm and calcifications. Visualization of the gallbladder wall is essential in differentiating between a positive wall-echo-shadow (WES) sign and a porcelain gallbladder. While a hypoechoic gallbladder wall is indicative of a WES sign, a hyperechoic wall layer will indicate a calcified gallbladder wall, suggesting a porcelain gallbladder. In image 1, the hyperechoic gallbladder wall can be visualized (white arrow), suggesting the presence of porcelain gallbladder and distinguishing it from a positive WES sign.

Computed tomography (CT) scan revealed cholelithiasis and porcelain gallbladder with associated moderate intrahepatic biliary duct dilation and a 1.5 cm hyperdensity within the distal cystic duct/common hepatic duct suggestive of choledocholithiasis. Image 2 is notable for the gallbladder and the calcifications (foci of bright white lines) in the gallbladder wall (white arrow). This image confirms the suspicion for porcelain gallbladder initially seen on bedside ultrasound.

## Patient course

The patient was admitted to the hospital with a gastroenterology consultation. The patient underwent stent placement in the stricture near the common hepatic duct. The patient was released from care and advised to follow up for elective outpatient cholecystectomy.

On follow-up, our patient was re-admitted to the hospital three months later, exhibiting sepsis due to infective endocarditis. On MRI of her head, the patient was found to have a septic embolus from endocarditis in the setting of bacteremia. The infectious disease team believes the bacteremia was caused by a biliary obstruction in the setting of gallbladder cancer. This is highly likely as the patient was diagnosed with PGB three months earlier.

## Discussion

When the inner gallbladder wall is covered with calcium, the wall becomes brittle and hardened with a bluish color. This can range from one calcified plaque attached to the wall to complete replacement of the gallbladder wall with calcium, also known as porcelain gallbladder (PGB).^6^ Patients presenting to the ED with PGB are often either asymptomatic or experience nonspecific symptoms, including abdominal pain, nausea, vomiting, fever, jaundice, or anorexia. Due to PGB's potential premalignant nature, it is vital to diagnose and treat PGB aggressively.^2^

Currently, PGB can be diagnosed by an abdominal x-ray exam, CT scan, abdominal US, or MRI.^7^ When a patient presents with symptoms of cholecystitis, most often PGB can be diagnosed with US. However, the sonographic appearance of PGB on ultrasound closely resembles WES sign, thus creating difficulty when trying to diagnose on POCUS.^8^ While there are reports of correctly diagnosing PBG through US, the use of POCUS by an emergency physician to diagnose PGB and differentiating between PGB and WES sign on US is unique.

Wall-echo-shadow (WES) sign on the ultrasound refers to the visualization of the gallbladder wall (GBW), the stone's Echo, and the acoustic Shadow. The appearance of WES sign on ultrasound indicates the patient's gallbladder lumen is filled with either one or multiple stones.^8^ WES sign demonstrates a contracted GBW, stones with clean shadows, and a portal triad. In comparison, porcelain gallbladder has an echogenic wall with some shadowing and scattered, irregular clumps of echoes. The irregular clumps of echo can be seen in Image 3 (blue arrow). When trying to differentiate between the two, it is helpful to determine whether a hypoechoic wall is present indicating WES sign or a calcified wall with no hypoechoic layer is present indicating PGB. If the results of the ultrasound are challenging to interpret, a radiology performed US or CT scan needs to be ordered and a gastroenterologist should be consulted.

Current treatment of PGB is typically a laparoscopic cholecystectomy in any symptomatic patient.^9^ However, due to the composition of PGB, it can be difficult to dissect and grasp the gallbladder.^2^ Patients with non-cancerous porcelain gallbladders who have undergone cholecystectomies have the same outcomes as those who undergo surgery for routine cholecystectomies.^6^ However, when a malignant gallbladder is found, stage one patients have a 50 percent five-year survival rate, stage two patients have 28 percent, stage three patients have eight percent, and stage four have two percent.^2^

We present a case of PGB being identified using POCUS. This case report explains the sonographic appearance of porcelain gallbladder and further disseminates the differences between PGB and WES signal which are often confused. Due to the possibility of PGB progression to cancer, it is crucial to diagnose PGB correctly, and refer the patient to a gastroenterologist and surgeon for elective cholecystectomy.

## Supplementary Information






