# Supplementary figures and images for: A Case Report on Detecting Porcelain Gallbladder form Wall-Echo-Shadow Sign on Point-of-Care Ultrasound
Source: J Educ Teach Emerg Med. 2021 Apr 19;6(2):V25–9. doi: 10.21980/J8164G (PMC10332777; doi:10.21980/J8164G)

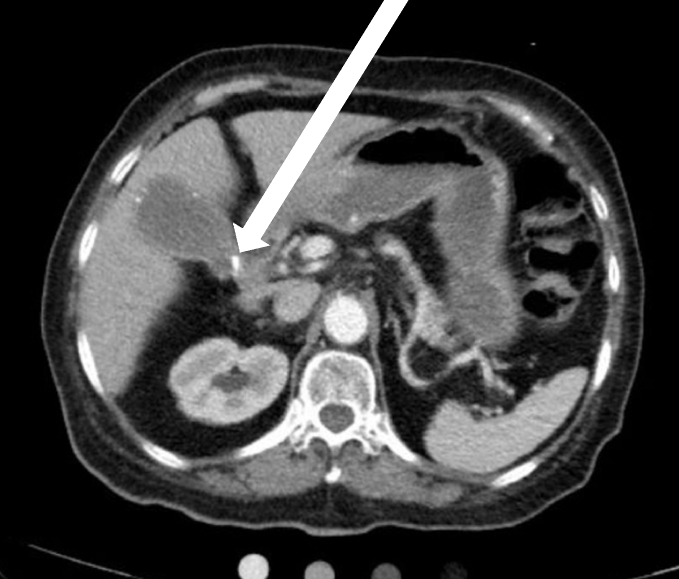

Supplement: Supplementary file 1 [file jetem-6-2-v25-supp1.jpeg]

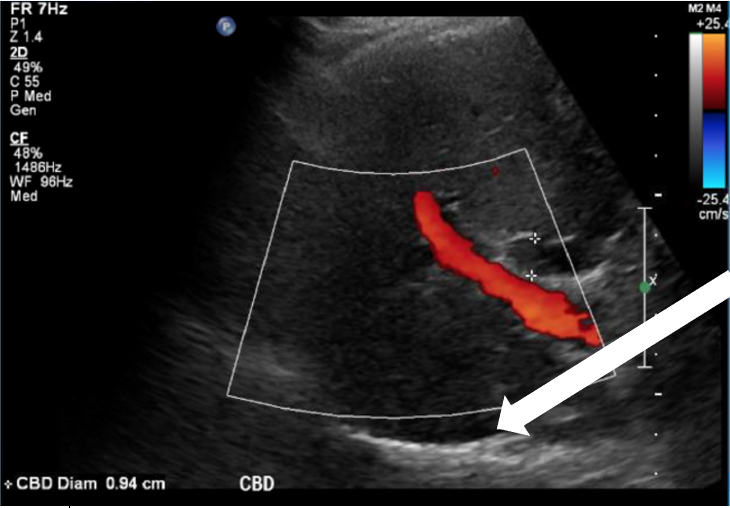

Supplement: Supplementary file 2 [file jetem-6-2-v25-supp2.jpeg]

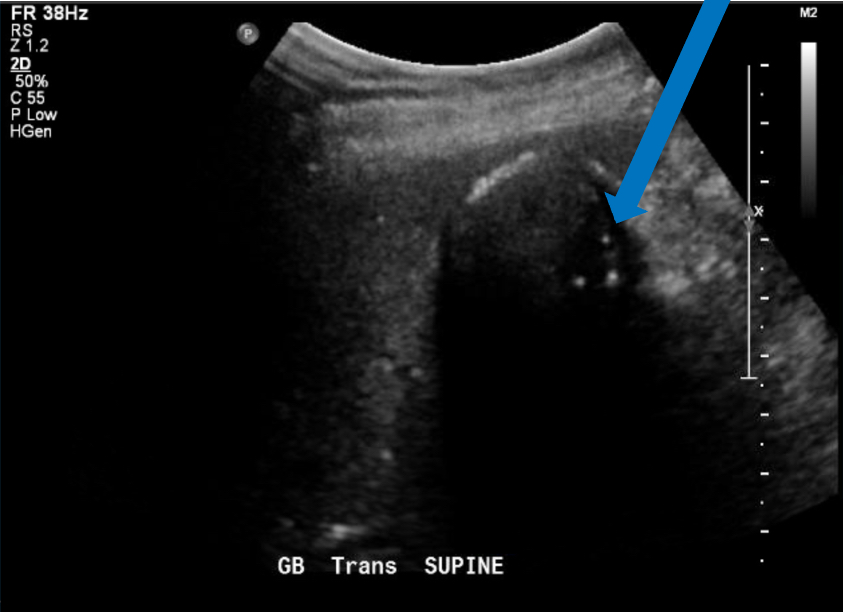

Supplement: Supplementary file 3 [file jetem-6-2-v25-supp3.jpeg]
